# Gut Microbiota and Intestinal Trans-Epithelial Permeability

**DOI:** 10.3390/ijms21176402

**Published:** 2020-09-03

**Authors:** Bénédicte Allam-Ndoul, Sophie Castonguay-Paradis, Alain Veilleux

**Affiliations:** 1 Centre Nutrition, Santé et Société (NUTRISS), Institut sur la Nutrition et les Aliments Fonctionnels (INAF), Université Laval, Québec, QC G1V 0A6, Canada; benedicte.allam-ndoul@criucpq.ulaval.ca (B.A.-N.); sophie.castonguay-paradis.1@ulaval.ca (S.C.-P.); 2 Canada Research Excellence Chair in the Microbiome-Endocannabinoidome Mediators Axis in Metabolic Health (CERC-MEND), Université Laval, Québec, QC G1V 0A6, Canada; 3 École de Nutrition, Faculté des Sciences de L’Agriculture et de L’Alimentation (FSAA), Université Laval, Québec, QC G1V 0A6, Canada

**Keywords:** intestinal epithelial organoids, small intestine, colon, gut microbiota, trans-epithelial permeability, tight junction

## Abstract

Constant remodeling of tight junctions to regulate trans-epithelial permeability is essential in maintaining intestinal barrier functions and thus preventing diffusion of small molecules and bacteria to host systemic circulation. Gut microbiota dysbiosis and dysfunctional gut barrier have been correlated to a large number of diseases such as obesity, type 2 diabetes and inflammatory bowel disease. This led to the hypothesis that gut bacteria-epithelial cell interactions are key regulators of epithelial permeability through the modulation of tight junctions. Nevertheless, the molecular basis of host-pathogen interactions remains unclear mostly due to the inability of most in vitro models to recreate the differentiated tissue structure and components observed in the normal intestinal epithelium. Recent advances have led to the development of a novel cellular model derived from intestinal epithelial stem cells, the so-called organoids, encompassing all epithelial cell types and reproducing physiological properties of the intestinal tissue. We summarize herein knowledge on molecular aspects of intestinal barrier functions and the involvement of gut bacteria-epithelial cell interactions. This review also focuses on epithelial organoids as a promising model for epithelial barrier functions to study molecular aspects of gut microbiota-host interaction.

## 1. Introduction

Mucosal surfaces, including the pulmonary tree, the genitourinary tract or the gastrointestinal tract, are covered by a layer of epithelial cells forming the interface of the body with the external environment [1]. The gastrointestinal mucosa is a semipermeable barrier allowing the absorption of nutrients, whole body homeostasis regulation and immune sensing, while limiting the passage of potentially harmful antigens and microorganisms from the intestinal lumen. This complex system includes, from the luminal to the basolateral surface, the gut microbiota, the mucus layer, the epithelial cell monolayer and immune cells in the lamina propria. The mucus layer and the epithelial cell monolayer act as a physical barrier, which forbade bacterial adhesion and regulate trans-epithelial diffusion of small molecules and bacteria to host systemic circulation. As for the lamina propria and the submucosa, they organize the immune response to face the passage of commensal and pathogenic microbes [2,3]. The regulation of trans-epithelial permeability is achieved by the tight junctions (TJ), which mechanically link epithelial cells together. Gut bacteria-epithelial cell interactions have been suggested as key contributors of TJ remodeling and so of epithelial permeability. Of note, dysregulation of the gut microbiota is tightly associated to the pathogenesis of several chronic diseases harboring intestinal permeability alterations, such as obesity, type 2 diabetes (T2D) and inflammatory bowel disease (IBD) [4,5,6]. This review proposes to briefly cover intestinal barrier components and functions in obesity and to explore bacteria-host interaction in the regulation of intestinal barrier function.

## 2. Components of the Intestinal Barrier

### 2.1. Gut Microbiota

The term gut microbiota refers to the microorganism community residing in the gut lumen. Adult gut microbiota harbors approximately 10^13^ bacterial cells [7] from more than 250 different species of bacteria as well as fungi, viruses and archaea. Human gut bacteria are mainly from the Firmicutes (60 to 80%), the Bacteroidetes (20 to 40%), the Proteobacteria and the Actinobacteria phylum [8,9,10,11] however, their relative abundances fluctuate according to the anatomical location and are highly variable among individuals [12]. Although gut microbiota composition can rapidly be altered by drugs, diet and other environmental factors, it is relatively resilient in the long term [13]. Host and gut microbiota communities co-exist in a highly mutualistic relationship that has crucial impacts on health status [14]. In fact, the host needs the gut microbiota to support diverse gut and systemic physiological functions, namely nutrient metabolism, maintenance of an optimal metabolic homeostasis and prevention of intestinal colonization by pathogenic bacteria.

### 2.2. Intestinal Mucosa

The intestinal mucosa is the innermost layer of the intestinal tract. It includes the epithelium, an underlying lamina propria of highly vascularized interstitial tissue, and the muscularis mucosae, which provide support and mobility to the mucosa [1,2]. The intestinal mucosa is overlaid with a discontinuous mucus layer that forms a highly organized glycoprotein network. Mucus is mainly composed of mucin proteins, especially Mucin 2, secreted by specialized epithelial cells called Goblet cells. This gel-like structure acts as a physical barrier, permeable to water and small molecules, limiting direct contact between the contents of the gut lumen and epithelial cells [15]. It also displays antimicrobial properties through the action of secreted antimicrobial peptides (i.e., defensins and IgA) but also represents an important bacterial niche. The thickness and the composition of the mucus layer influences the properties of this bacterial niche, whilst the bacteria can also impact the properties of the mucus layer [16].

The intestinal epithelium is composed of a polarized cell monolayer of absorptive enterocytes, Paneth cells, Goblet cells and enteroendocrine cells. It is the most crucial component of the physical intestinal barrier separating the lamina propria from the lumen [3]. Junctions between epithelial cells, established by the adherens junctions, must be strong but malleable to cope with the movements and stretches of the intestinal tract. These junctions are also crucial to maintain epithelium polarity, which allows the directional transport of nutrients and the secretion of enzymes and antimicrobial peptides into the lumen.

### 2.3. Tight Junctions

Tight junctions consist of a network of transmembrane protein strands that interacting and link laterally adjacent cells near the apical surface of the epithelium [17]. These transmembrane proteins include large families of claudin proteins, TJ-associated MARVEL domain-containing proteins (TAMPs) and junctional adhesion molecules (JAMs) as well as a variety of cytoplasmic adaptor and scaffolding proteins [18].

Both *cis*- and *trans*-homophilic interactions of claudin proteins within the transcellular space create a barrier or pores enabling selective ion permeability and thus are major determinants of *trans*-epithelial transport [19]. At least 27 members of the claudin family have been identified, some with barrier functions for small molecules and others, such as claudin-2, with pore-forming properties for ions and water [20]. Of note, the knockout of claudin 7 [21] or claudin 2 and 15 [22] members in mice caused intestinal barrier disturbances. The TAMP protein family, i.e., occludins, tricellulin and MarvelD3, are mainly involved in *cis*-homophilic interactions along the cell membrane. Their presence appears as essential for TJ formation and maintenance; however, their precise roles remain unclear [23] Single-span transmembrane protein members of the JAM family form a multitude of intracellular and extracellular molecular interactions, which regulate TJ assembly and functions [24]. Combination and spatial layout of these proteins allow tissue-specific TJ permeability as well as dynamic regulation of the TJ properties to adapt to changing environmental conditions such as diet and microbiota.

TJ proteins are linked to the actin-based cytoskeleton through adaptor and scaffolding molecules. These interactions are crucial to maintain gut epithelial integrity by detecting mechanical stress and by coordinating changes in protein expression and assembly accordingly [25]. Scaffolding proteins belonging to the zonula occludens family (ZO-1, ZO-2 and ZO-3) are the best-known multidomain proteins. ZO-1 is essential in coordinating TJ formation and cell polarization as it establishes links between most transmembrane TJ proteins (i.e., claudins, TAMPs and JAMs) and cytoskeleton components [26].

These complex multiprotein structures create an essential barrier against potentially harmful pathogens and molecules in the lumen. TJ are highly dynamic and can adapt to the changing environment. However, environmental, and cellular events may partially or completely disrupt the intestinal barrier leading to chronically increased intestinal permeability and allowing intrusion of harmful environmental components into systemic circulation. A dysfunctional barrier is a common feature of obesity and T2D [27].

### 2.4. Intestinal Barrier Function in Obesity and T2D

Obesity is an important global health problem, which is associated with increased risk of cardiometabolic complications, such as T2D [28]. These conditions share low-grade inflammation as well as alterations in the intestinal barrier function. The compromised epithelial barrier arises from changes in TJ protein expression or modifications of key intracellular and extracellular molecular interactions facilitating the passage of potentially harmful bacterial antigens and microorganisms from the intestinal lumen to systemic circulation [29].

Pro-inflammatory lipopolysaccharide (LPS) from some Gram-negative bacteria has been identified as a key contributing factor in the initiation and the progression of low-grade inflammation in presence of leaky gut [27]. Under normal conditions, the intestinal epithelium prevents LPS translocation, but in diet-induced obesity (DIO) as well as in obesity and T2D mice model (*db*/*db*) higher circulating LPS levels and low-grade inflammation in peripheral tissues are noted [30]. Moreover compared to lean control mice, DIO and *db*/*db* mice showed a higher trans-epithelial permeability and a modified distribution of ZO-1 in the intestinal mucosa [31]. Such alteration in gut permeability is accompanied by a decreased expression of several TJ proteins, i.e., ZO-1, occludin [32,33]. In humans, upper gastrointestinal tract trans-epithelial permeability, measured by urinary sucrose recovery, is elevated in presence of obesity [34]. However, obesity may not be the most critical determinant of intestinal barrier dysfunction as hyperglycemia strongly interfered with intestinal barrier integrity (i.e., decrease ZO-1 mucosal staining and increased fluorescent-dextran absorption) in obese and T2D mice [35]. Hyperglycemia was established as a direct cause of intestinal barrier dysfunction as treatment of this condition, but not obesity, rescued glucose-induced barrier alterations in mice [35]. Similarly, inadequate glycemic control in humans has been associated with increased translocation of microbial products in the circulation [35]. Intestinal permeability is also increased along with the severity of liver steatosis in obese subjects [31,36]. These demonstrations underline that obesity-related complications are closely associated with altered intestinal barrier functions. Although the precise mechanisms linking metabolic complications to intestinal permeability remain unclear, the literature suggests an active role of the gut microbiota.

### 2.5. Gut Microbiota Dysbiosis

Gut bacterial communities have a high degree of plasticity and their composition can be affected by numerous environmental and host factors such as diet, age, physiological state and genetic background. Gut dysbiosis, characterized by an imbalance in the composition and activity of gut microbial communities, has been associated with the development of several diseases [5,37]. Fecal microbiota transfer and germ-free animal studies have established a causal link between gut microbiota dysbiosis and diseases such as obesity and enteropathies [38,39]. Changes in the metabolic activity of gut microbiota, such as its capacity to harvest energy from nutrients, may contribute to the establishment of these diseases [40] but the role of more complex gut microbiota-host interactions are being explored.

It is often acknowledged that the relative abundance of the two main gut microbiota phyla, i.e., Bacteroidetes and Firmicutes, is altered in obesity such that the Firmicutes-to-Bacteroidetes ratio is higher in obese subjects [41,42]. In fact, some studies found that Bacteroidetes are positively associated with leanness and weight loss, whereas Firmicutes and Proteobacteria are generally positively associated with low quality diet, obesity and metabolic complications [43,44]. Even though some of these findings have been recently challenged, the contribution of gut microbiota in obesity-related complications is undeniable and far more complex than a simple imbalance in phylum relative abundance [45,46].

The impact of specific genera and species within these microbial phyla are not necessarily consistent. Indeed, the *Lactobacillus* genus, belonging to the Firmicutes phylum, has been associated with obesity and T2D [47] while some species of this genus, i.e., *Lactobacillus paracasei* and *Lactobacillus plantarum* appear to have a protective effect against weight gain [48,49]. Obesity and T2D have been associated with a lower relative abundance of *Bacteroides* and *Bifidobacterium* genera as well as butyrate-producing bacteria *Faecalibacterium prausnitzii* [50,51] and *Roseburia intestinalis* genera [52]. Interestingly, bariatric surgery increased *F. prausnitzii* relative abundance concomitantly with an amelioration of glucose homeostasis, low-grade inflammation and gut epithelial permeability in subjects with T2D [53,54]. This species is one of the main butyrate-producing bacteria, a key energy source for intestinal epithelial cells [55]. Butyrate has been shown to reduce local inflammation and to improve gut barrier permeability through several mechanisms among which mucin synthesis [56], TJ reassembly [57] or occludin and ZO-1 up-regulation [58]. *Bacteroides vulgatus* and *Bacteroides dorei*, two species potentially beneficial for T2D, have been shown to increase the expression of ZO-1 and to improve epithelial barrier function [59]. These bacteria can produce bacteriocins, proteins that inhibit growth of specific bacteria, which could participate in the development or reversal of dysbiosis by limiting the growth of harmful strains [60]. *Akkermansia muciniphila,* a mucin-degrading strain belonging to the Verrucomicrobia phylum, colonizes the intestinal mucus layer where it ameliorated intestinal barrier integrity directly by promoting mucin production [61] and indirectly through interactions with other bacteria [62,63]. This species has consistently been associated with leanness, insulin sensitivity and lessened low-grade inflammation [62,64]. Interestingly, either pasteurized or live *A. muciniphila* were able to reduce gut barrier permeability and to improve glucose metabolism in obese mice [64,65]. Finally, the relative abundance of the main LPS-producing Gram-negative bacteria is modified in obesity. Indeed, imbalanced or dysbiotic gut microbiota tends to shift away from less potent Bacteroidetes LPS in favor of more pro-inflammatory Proteobacteria LPS [66,67]. Increased pro-inflammatory endotoxin levels in dysbiotic gut microbiota may in turn exacerbate the low-grade inflammatory state by increasing trans-epithelial permeability, which facilitates translocation of even more pro-inflammatory LPS into the circulation [63,68].

A tripartite interrelation between metabolic complications, intestinal barrier function and gut bacteria seems to emerge from recent literature. Environmental and host diseases-related factors impact both gut microbiota composition and intestinal barrier integrity. As described, bacteria and gut microbiota-derived factors are also direct modulators of the intestinal barrier integrity. Yet, the precise molecular bases of gut microbiota-host interaction remain unclear. Obtaining comprehensive knowledge from relevant models of intestinal barrier function will be crucial to establish mechanisms of interactions to develop strategies to resolve epithelial barrier dysfunctions and alleviate ensuing metabolic complications.

### 2.6. Bacteria-Host Interaction and Intestinal Barrier Function

#### 2.6.1. Intestinal Epithelial Organoids

Immortalized epithelial cell lines (e.g., Caco-2, HT-29 and T84) have permitted breakthrough in gut mucosal physiology for decades but have several weaknesses and limitations [69,70]. Recent advances have led to the development of a novel cellular model derived from intestinal epithelial stem cells, the so-called epithelial organoids. These organoids are three-dimensional self-renewing cellular structures embedded in extracellular matrix maintaining their organ-specific cell lineage [71]. Intestinal epithelial organoids reproduce villus-like structures and crypt-like proliferative zones, which encompass all epithelial cell types [72]. Intestinal epithelial organoids can be derived from freshly isolated intestinal crypts holding multipotent adult stem cells (ASCs) or from pluripotent stem cells (PSCs) [71]. PSCs emanate either from embryonic stem cells (ESCs) or from patient somatic cells which undergo reprogramming into a pluripotent state (iPSCs) [73]. These cultures a remarkably stable, both phenotypically and genetically. Intestinal organoids have thus rapidly provided novel knowledge on the pathophysiology of several enteropathies [74,75]. Interestingly, these polarized epithelial cells establish functional TJ complexes [71,76] and secrete mucus onto the apical surface of the epithelium [77,78], which confirms the added value of using epithelial organoids as a model to study epithelial barrier functions in the gut.

Most anaerobic bacteria in the gut microbiota are not viable in the aerobic environment needed to harvest intestinal cell lines, limiting interaction studies to bacterial lysates and conditioned media rather than living bacteria. This limitation can be overcome using intestinal epithelial organoids as their intraluminal space is sealed within the mucus layer, the epithelium and the extracellular matrix, which allows for the establishment of slight hypoxia in the lumen (i.e., 5 to 15% of oxygen) [79]. Furthermore, epithelial organoids include, by definition, only epithelial cells. The absence of immune and stromal cells provides the opportunity to investigate direct bacteria-epithelial cell interactions.

Microinjection methods are needed to inoculate living aerotolerant or anaerobic bacteria in the organoid lumen for a short period of time [79]. Studies using monoculture and complex bacterial communities resulted in stable colonization to investigate gut bacteria-epithelial cell interaction [78,80,81]. While this approach is valuable and is the most widely used, it remains challenging due to equipment and skills required for microinjection in organoid lumen (<250 μm). Alternative monolayer culture methods allowing easy access to the apical surface of the epithelium were developed for large-scale experiments. Monolayer and three-dimensional cultures are thus complementary and together represent promising avenues to study cellular and molecular aspects of gut bacteria-epithelial cell interactions.

The next section reviews studies in which gut microbiota-host interactions were investigated using intestinal epithelial organoids to identify the cellular and molecular mechanisms involved in the regulation of epithelial barrier functions by gut bacteria. Table 1 details such studies. The use of bacterial and viral infections in intestinal epithelial organoids described below can be used as a model that could, at least in part, reflect gut microbiota-host interactions present in states of obesity-related gut dysbiosis.

#### 2.6.2. *Clostridium difficile*

*Clostridium difficile* is an anaerobic, Gram-positive, toxin-producing bacillus which is a major infectious cause of nosocomial diarrhea [82]. The virulence of this emerging pathogen is mostly conferred by two large proteins: *C. difficile* toxins A (TcdA) and *C. difficile* toxin B (TcdB) [83]. Both toxins have potent glucosyltransferase activity leading to cytotoxicity.

Viable C. *difficile* were microinjected into the lumen of intestinal epithelial organoids and able to persist for at least 12 h [79]. This illustrates the capacity of intestinal epithelial organoids to create a slightly anaerobic niche allowing the culture of anaerobes, at least for a short period of time. Colonization of the epithelial organoids with a toxigenic *C. difficile* strain triggered a complete disruption of the epithelium. Loss of epithelial barrier function was accompanied by an important redistribution of the TJ proteins ZO-1 and occludin as well as an important down-regulation of mucin 2 expression [78]. All these effects were reproduced by microinjection of TcdA, but not of TcdB, in organoids.

*C*. *difficile* infection is also associated with increased mucosal levels of pro-inflammatory cytokines (i.e., IFN-γ and TNF-α), which are capable of disrupting TJ and epithelial integrity in organoids as for *C*. *difficile* toxins [96]. Host STAT5-dependent JAK2 signaling pathway is necessary to initiate the production of anti-inflammatory cytokines and to promote intestinal epithelium repair. By taking advantage of genetically modified organoids, Liu et al. demonstrated the involvement of a defective JAK2-STAT5 signaling in the susceptibility to *C*. *difficile* infection. Indeed, constitutive activation of this pathway is sufficient to restore the expression of TJ proteins and to maintain epithelium integrity in organoids exposed to pro-inflammatory cytokines or C. difficile toxins [96].

In summary, these results demonstrate that viable bacteria can persist in the lumen of intestinal epithelial organoids and that such coculture models can be valuable in the mechanistic study of interactions between gut bacteria and intestinal epithelial barrier functions. Importantly, three-dimensional intestinal organoids display a physiologically relevant response to *C*. *difficile* colonization, which facilitates knowledge translation of the observed molecular and cellular events to in vivo situations.

#### 2.6.3. *Escherichia coli*

Shiga toxin (Stx)-producing *Escherichia coli*, including the O157:H7 strain, are involved in the development of acute bloody diarrheal diseases and are commonly associated with foodborne outbreaks [97]. Developing effective interventions for diseases caused by Stx-producing *E. coli* is of importance as such infections can lead to the development of life-threatening systemic complications such as hemolytic uremic syndrome and kidney failure [98]. Until now, research lacked effective experimental models to properly investigate human-restricted pathogens such as Shiga toxin-producing *E. coli*.

Pradhan and al. investigated the impact of two major forms of Shiga toxins, i.e., Stx1 and Stx2a, on gut barrier function using intestinal epithelial organoids [86]. Significant dose- and time-dependent increases in trans-epithelial permeability were observed in organoids exposed to both types of Stx toxins, either in the luminal and the basolateral compartments. Microinjection of the more potent toxin, Stx2a, in human intestinal organoids triggered a paradoxical up-regulation of several key structural and TJ proteins, i.e., ZO-2, occludin and claudin-1, as well as mucin 2 expression. To assess susceptibility to Stx in vivo, human intestinal epithelial organoids were transplanted under the kidney capsule of mice for two months, where they developed large mucous-filled lumens with native crypt-villus architecture. Injection of Stx2a in the lumen of in vivo organoids caused blood accumulation in the villi and mesenchyme of the transplants, epithelial damages and apoptosis [85].

In another study, human colonic epithelial organoids were cultured as monolayers to investigate Shiga toxin-producing *E. coli*-epithelial cell interactions [87]. Four hours post-infection, mucin layer thickness and mucin 2 labelling were significantly reduced, which allowed *E. coli* to directly interact with the epithelium. Within 18 h, the infection induced an important redistribution of occludin from the TJ complexes to the cytosol and a concomitant decrease of the trans-epithelial electrical resistance, a marker of altered epithelial permeability. Thus, human intestinal epithelial organoids, either as a monolayer, matrix-embedded or transplanted in mice, represent an interesting model to investigate cellular and molecular aspects of pathogen-host interactions especially in the context of intestinal barrier function.

#### 2.6.4. *Lactobacillus rhamnosus* GG

Probiotics are currently defined as “live microorganisms that, when administered in adequate amounts confer a health benefit on the host” [99]. The beneficial role of probiotics relies on their ability to impede gut pathogen colonization, to modulate host immune response and to enhance the intestinal epithelial functions, among others [99,100].

*Lactobacillus rhamnosus GG* (LGG) is a probiotic originally isolated from the intestinal tract of a healthy subject, which has been extensively used for its various health benefits. It has clinically been shown to improve IBD symptoms and to maintain remission of ulcerative colitis [101]. Using organoids, Han et al. investigated the protective role of LGG on epithelial barrier functions by incubating human intestinal epithelial organoids with fecal supernatants of IBD patients or healthy subjects in the presence or absence of LGG [88]. Intriguingly, fecal supernatants from IBD patients, but not from healthy subjects, increased trans-epithelial permeability only in the absence of LGG supplementation. LGG conditioned media restored epithelial barrier functions after a barrier disruption by pro-inflammatory cytokines by normalizing TJ protein expressions, especially ZO-1 and occludin. Interestingly, only secreted metabolites, and not bacterial cell wall or bacterial DNA, were able to prevent IFN-γ-induced epithelial damages.

Identification of soluble factors mediating the effects of probiotics, or more generally of gut bacteria, represent an opportunity to understand their mechanism of action as well as to develop effective pharmacological strategies to circumvent issues posed using live bacteria. This study showed the relevance of using intestinal epithelial organoid as a simplified, but highly relevant, in vitro model to unravel the mechanisms involved in bacterial-epithelial cell interactions.

#### 2.6.5. Viral Infections

Viral infection is the most frequent cause of gastroenteritis in humans, being involved in more than 60% of diarrhea symptoms [102]. Norovirus and rotavirus are highly prevalent in the gastrointestinal tract, which spread through contaminated foods, surfaces or fecal-oral transmission. Viral replication takes place within the gut lumen and requires direct interaction with the intestinal epithelium [103]. There is, to our knowledge, no study investigating the impact of viral infection on intestinal barrier functions in organoid models. Nevertheless, it has been shown that intestinal epithelial organoids are an excellent in vitro model to study rotavirus infections.

Absence of pharmaceutical prevention or treatment strategies for some virus, such as human noroviruses (HuNoVs) is partly due to the lack of relevant in vivo and in vitro infection models. Ettayebi et al. exploited human intestinal epithelial organoids as a monolayer culture to demonstrate that HuNoVs can replicate in vitro [104]. Interestingly, these cultures also recapitulate genotype-specific patterns of HuNoV susceptibility as cell cultures with the secretor positive genotype (Fucosyltransferase 2, FUT2) were more susceptible to viral infections than cultures isolated from secretor negative individuals. This observation mirrors epidemiologic data suggesting that HuNoV infection is dependent on the expression of genetically determined histoblood groups. Similarly, Yin and al. demonstrated the ability of murine and human organoids to support rotavirus infections [105]. Indeed, SA11 rotavirus replicons were detected in mice and human epithelial organoids 24 h post-incubation. Viral infection was associated with increased INF-γ-mediated innate immune response of host cells.

These studies suggest that it is conceivable to take advantage of intestinal epithelial organoids to investigate the impact of viruses on intestinal barrier functions. Patient-derived organoids can be used to study the pathogenesis of gastroenteritis and to support development of new therapeutics. Use of such organoids also revealed that the genetic and pathophysiological background of the stem cell donors must be taken into consideration but can also represent a promising avenue to address interindividual variability in gut microbiota-host interactions.

## 3. Conclusions

Alterations in gut epithelial permeability and gut microbiota dysfunction are both tightly associated to the pathogenesis of several chronic diseases, such as obesity and type 2 diabetes (T2D). Gut bacteria-epithelial cell interactions have been suggested as a key contributor of epithelial permeability in several segments of the gastrointestinal tract. In fact, several studies support the notion that bacteria can regulate TJ expression and assembly, and thus regulate trans-epithelial permeability. This review has discussed opportunities of using intestinal organoid as in vitro study models of gut microbiota-epithelial cell interactions. While most studies have focused on human pathogens, the use of bacterial and viral infections in intestinal epithelial organoids represents an excellent opportunity to study the interactions of individual species or more complex bacterial communities with gut physiology in states of gut dysbiosis. Comprehensive knowledge about the role of the gut microbiota on intestinal barrier function from a highly relevant model, i.e., epithelial organoids, will be crucial in developing strategies to resolve epithelial barrier dysfunctions in several non-infectious chronic diseases.

## Figures and Tables

**Table 1:** Studies reporting gut microbiota-epithelial cell interactions using intestinal epithelial organoids.

Bacteria and Viruses	Strain and Type	Host and Segment	Culture Method	Study Outcome(s)	Ref
*Clostridium difficile*	1870	Human iPSC	Matrix-embedded	↓ mucin production (mucin 2)	[78]
	VPII 10463	Human iPSC	Matrix-embedded	↑ trans-epithelial permeability (TcdA toxin > TcdB toxin) Alteration of ZO-1 and occludin expression	[79]
*Cryptosporidium parvum*	―	Mouse Adult stem cells	Matrix-embedded	↓ epithelial cell growth	[84]
*Escherichia coli*	Non-pathogenic ECOR2	Human ESC	Matrix-embedded	Colonization of the organoid lumen with *E. coli* Regulates adherents junctions and other cell-cell interactions ↑ epithelial mucus secretion. ↓ IFN-γ-induced trans-epithelial permeability	[81]
	Shiga toxin-producing O157:H7	Human iPSC	Matrix-embedded	↓ epithelial barrier integrity	[85]
	Shiga toxin-producing O157:H7	Mouse iPSC	Matrix-embedded	↑ TJ proteins (TJ protein 2, occludin and claudin-1) and mucin proteins ↑ trans-epithelial permeability	[86]
	Enterohemorrhagic EDL933	Human ASC Colon	Monolayer	↓ mucus production (mucin 2) ↓ protocadherin 24 protein and TJ proteins (Occludin).	[87]
*Lactobacillus*	*L. rhamnossus GG*	Human ASC small intestine	Matrix-embedded	↑ occludin and ZO-1 expression ↓ IFN-γ-induced trans-epithelial permeability ()	[88]
	*L*. *reuteri* D8	Mouse ASC small intestine	Matrix-embedded	↑ intestinal epithelial cell proliferation Decreases trans-epithelial permeability	[89]
*Salmonella entetica*	*Serovar Typhimurium* 14028	Mouse ASC small intestine	Matrix-embedded	↓ TJ complexes and TJ proteins (ZO-1 and occludin) ↑ claudin-2 protein	[90]
	*Serovar typhimurium* SL1344	Human iPSC	Matrix-embedded	Invades organoids epithelial barrier	[91]
*Shigella flexneri*	2457T	Human ASC small intestine	Monolayer	↑ mucus production (Muc2)	[92]
Enteroviruses	CVB3, EV-71, E11	Human ESC	Matrix-embedded	↑ cytotoxicity (Enterovirus E11) ↑ misclocalization of occludin ↓ crypt morphology and integrity	[93]
	A71	Human ESC	Monolayer	↑ trans-epithelial permeability	[94]
	A71	Human ESC	Monolayer	↓ Mucin-1 and 2 production No impact on epithelial barrier function	[95]

iPSC: Induced pluripotent stem cells; ECS: Embryonic stem cells; ASC: Adult stem cells.
